# Strongly reduced lattice thermal conductivity in Sn-doped rare-earth (M) filled skutterudites M_*x*_Co_4_Sb_12−*y*_Sn_*y*_, promoted by Sb–Sn disordering and phase segregation†

**DOI:** 10.1039/d1ra04270j

**Published:** 2021-08-03

**Authors:** J. Gainza, F. Serrano-Sánchez, N. M. Nemes, O. J. Dura, J. L. Martínez, F. Fauth, J. A. Alonso

**Affiliations:** Instituto de Ciencia de Materiales de Madrid (ICMM), Consejo Superior de Investigaciones Científicas (CSIC), Sor Juana Inés de la Cruz 3 E-28049 Madrid Spain j.gainza@csic.es ja.alonso@icmm.csic.es; Departamento de Física de Materiales, Universidad Complutense de Madrid E-28040 Madrid Spain; Departamento de Física Aplicada, Universidad de Castilla-La Mancha Ciudad Real E-13071 Spain; CELLS–ALBA Synchrotron Cerdanyola del Valles Barcelona E-08290 Spain

## Abstract

CoSb_3_ thermoelectric skutterudite has been filled with rare-earth metals (M = La, Ce, Yb) and partially doped with Sn in specimens of M_*x*_Co_4_Sb_12−*y*_Sn_*y*_ stoichiometry. This has been achieved under high-pressure conditions at 3.5 GPa in a piston-cylinder hydrostatic press. A structural investigation using synchrotron X-ray diffraction data reveals a phase segregation in twin skutterudite phases with filling fraction fluctuation and different unit-cell sizes. As a result of three effects acting as phonon scatterers, namely the rattling effect of M at the wide 8*a* cages of the cubic *Im*3̄ structure, the phase segregation, and the intrinsic disorder introduced by Sn substitution at the Sb sublattice, the total thermal conductivity (*κ*) dramatically falls to reach minimum values under 2 W m^−1^ K^−1^, well below those typically exhibited by other thermoelectric materials based upon single-filled skutterudites. The power factor is substantially enhanced to 1.11 mW m^−1^ K^−2^ in Yb_0.5_Co_4_Sb_11.6_Sn_0.4_ with respect to the unfilled composition, as a result of the charge transfer promoted by the filler.

## Abstract 2

Three effects act as phonon scatterers: rattling of M at 8*a* cages, phase segregation, and intrinsic disorder introduced by Sn substitution, resulting in minimum *k* values. High filling fraction in Sn-doped phases pave the way to tune the carrier density in skutterudites.

## Graphical abstract

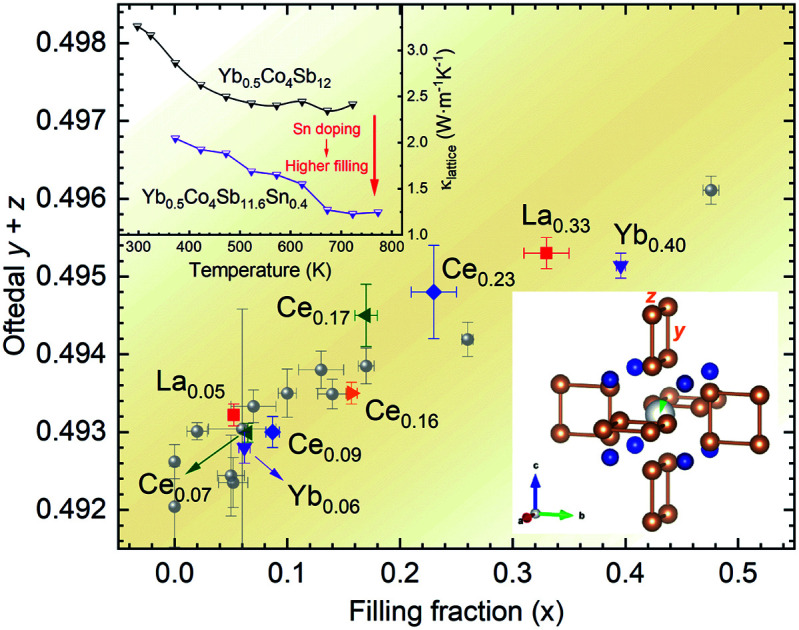

## Introduction

With the world facing an unstoppable growth in energy needs, the interest in alternative energy sources becomes greater every day. Thermoelectric materials, with the ability to convert heat into electricity, can be a good choice to help overcome this problem. The efficiency of these materials can be evaluated with the dimensionless figure of merit,^1,2^ ZT = *S*^2^*T*/*ρκ*_t_, where *S*, *T*, *ρ* and *κ*_t_ are the Seebeck coefficient, absolute temperature, electrical resistivity and total thermal conductivity, respectively. This total thermal conductivity is the sum of the electronic and lattice contributions. Although thermoelectrics have been used for half a century, they have found only very specific applications due to their relatively low efficiency, with ZT in commercial materials limited to around 1, and economically feasible use requiring ZT above 3.^3^ However, with a resurgent research activity in recent years, thermoelectric materials have seen their performance greatly improve with ZT values reaching beyond 2.^4–6^

Some of the most promising materials are those based on skutterudites, more precisely, those based on cobalt and antimony,^7–9^ of CoSb_3_ stoichiometry with skutterudite crystal structure. It essentially consists of a cubic framework of tilted CoSb_6_ octahedra sharing corners. Although the thermal conductivity of the pure skutterudite is high, its doped compounds exhibit crystalline electronic transport combined with glass-like thermal phonon transport, making them good candidates for thermoelectric applications. In addition, many guest atoms,^10–12^ such as rare-earths,^13–16^ can be incorporated into the structural voids between the octahedra, in materials of M_*x*_Co_4_Sb_12_ stoichiometry (M = filler element, with *x* = 1 corresponding to complete site-filling, although *x* ≈ 0.5 seems to be the practical limit^17,18^), tuning the carrier density and reducing the intrinsically high lattice thermal conductivity, which is the main drawback in the thermoelectric performance of the unfilled skutterudite. The Wiedemann–Franz law connects the electrical conductivity and the electronic thermal conductivity, so a drastic reduction in the lattice thermal conductivity is a key requirement to develop successful thermoelectrics.^19^

Besides the lattice thermal conductivity, another key parameter for a thermoelectric is the electronic mobility. Doping at the Co sites^20,21^ hampers the electronic mobility more significantly than doping at the Sb sites,^22^ since the Co d orbitals are the main contributors to the skutterudite conduction bands.^23^ Bearing this in mind, doping at the Sb site seems better to improve the figure of merit, by means of keeping the mobility as high as possible while reducing the lattice thermal conductivity. Some examples for this approach use dopant elements such as Sn,^24,25^ Te,^26^ or even Ga.^22^

Recently, we have reported on the high-pressure synthesis and characterization of the unfilled CoSb_3_ (ref. 27) and some filled skutterudites,^28^ such as La_*x*_Co_4_Sb_12,_^29^ Ce_*x*_Co_4_Sb_12_,^30^ Yb_*x*_Co_4_Sb_12_,^30^ and Mm_*x*_Co_4_Sb_12_,^31^ (Mm: mischmetal = Ce + La) showing low thermal conductivities due to uneven distribution of rare-earth elements promoted by the high pressure synthesis. Here, we describe the synthesis and characterization of novel materials where Sb has partially been replaced by Sn, in samples of La_0.5_-, Ce_0.5_-, Yb_0.5_-Co_4_Sb_12−*y*_Sn_*y*_ stoichiometry accessed by high-pressure methods; we choose Sn to replace Sb since it is a p-block element, with a lone electron pair, which may adopt a similar coordination environment in the skutterudite crystal structure. Moreover, doping with Sn may enable higher filling fractions by rare-earth elements so as to compensate its acceptor doping. We focus primarily on the Ce-filled skutterudite in an attempt to improve its experimental filling fraction, which is reported to be rather low, around 0.10–0.15, a hindrance towards increased carrier concentration.^14^ These compounds also show the filling fraction fluctuation, due to a heterogeneous distribution of the filler elements. This phase segregation adds to the intrinsic disorder introduced by the Sn-doping at Sb positions, and to the higher filling fractions, and therefore we observe a noticeable reduction in the total thermal conductivity, from 3.4 W m^−1^ K^−1^ in Yb_0.5_Co_4_Sb_12_, to 2.4 W m^−1^ K^−1^ in Yb_0.5_Co_4_Sb_11.6_Sn_0.4_ close to RT, yielding a relatively high figure of merit of ∼0.4 for this very composition. Moreover, we empirically obtain from SXRD data refinement a high filling fraction of 0.23(2) for the Ce-filled compound in the majority phase, improving the previously reported data^14^ and paving the way to tune more conveniently the carrier density in this type of materials.

## Experimental

All the samples with different nominal compositions R_0.5_Co_4_Sb_11.6_Sn_0.4_ (M = La, Ce and Yb) and Ce_0.5_Co_4_Sb_12−*y*_Sn_*y*_, (with *y* = 0.2, 0.4, 1) have been prepared by a solid-state reaction at moderate temperature and under high pressure. The reagents used were La (99.9%, Strem Chemicals), Ce (99.9%, Alfa Aesar), Yb (99.9%, Alfa Aesar), Co (99%, ROC/RIC), Sb (99.5%, Alfa Aesar) and Sn (99.8%, Alfa Aesar) in powder form. About 1.1 g of the starting elements were mixed according to the stoichiometric amount and sealed in a niobium capsule in a N_2_-filled glove box, which was then placed inside pyrex tube that acted as hydrostatic pressure medium when melted by a graphite cylinder heater. Reactions were carried out in a piston-cylinder press (Rockland Research Co.), at a pressure of 3.5 GPa at 1073 K for 1 h. Afterwards, the products were quenched, and the pressure was released. The samples were obtained as circular pellets of ∼5 mm diameter, with a density around 90% of the theoretical crystallographic value. Some pellets obtained after the reaction were ground to powder for structural characterization and some were cut and polished for transport measurements.

Preliminary phase characterization was carried out using X-ray diffraction on a Bruker-AXS D8 diffractometer (40 kV, 30 mA), run by DIFFRACTPLUS software, in Bragg–Brentano reflection geometry with Cu Kα radiation (*λ* = 1.5418 Å). High-angular resolution experiments of synchrotron X-ray powder diffraction (SXRD) were performed in the MSPD beamline of the ALBA Synchrotron (Barcelona, Spain). The SXRD patterns were collected in the high-resolution MAD set-up^32^ with 28 keV beam energy (*λ* = 0.4427 Å). The samples were contained in rotating quartz capillaries of 0.7 mm diameter. SXRD patterns were collected at room temperature (RT, 298 K), 473, 673, 873 and 1073 K for the temperature-dependent analysis.

Both laboratory and synchrotron XRD data were analyzed by Rietveld refinement using the FullProf program.^33^ The peak shape was described using a pseudo-Voigt function. The full refinement included the following parameters: scale factors, zero-point error, background coefficients, asymmetry correction factors, lattice parameters, atomic positions, occupancy factors of the filler element, and anisotropic displacement parameters. The Sn/Sb doping concentration was fixed to the nominal value.

The Seebeck coefficient was measured using an MMR technologies instrument under vacuum (10^−3^ mbar) in the temperature range of 300–800 K. Conventional van der Pauw geometry was employed to determine the electrical resistivity. The total thermal conductivity was calculated from the thermal diffusivity (α) using a Linseis LFA 1000 equipment, by the laser-flash technique. The thermal conductivity (*κ*) is determined from *κ* = *αc*_p_*d*, where *c*_p_ is the specific heat, calculated using the Dulong–Petit equation, and *d* is the sample density.

## Results

### Structural characterization

**Fig. 1:**
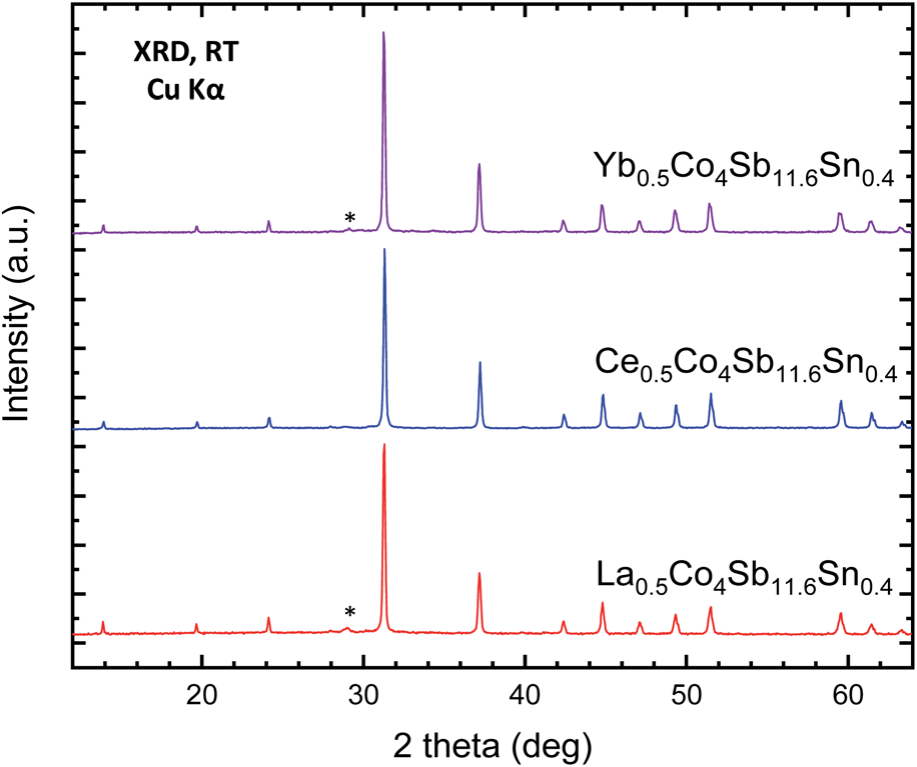
Laboratory XRD patterns of as-grown M_*x*_Co_4_Sb_11.6_Sn_0.4_ (M = Yb, Ce, La) at room temperature. The star corresponds to the most intense reflection of Sb impurity.

High-pressure synthesis conditions were essential to stabilize the skutterudite materials in short periods of time (typically 1 h), avoiding the oxidation of the metal elements (Sb, Sn) and in particular the rare-earth fillers (M). The resulting materials, referred to in this manuscript by their nominal filler composition M_0.5_Co_4_Sb_12−*y*_Sn_*y*_ (M = Ce, La, Yb), were initially characterized by laboratory X-ray diffraction (XRD). XRD patterns of representative samples for the different rare-earth fillers are displayed in Fig. 1. The laboratory XRD patterns of the whole series of Ce_0.5_CoSb_12−*y*_Sn_*y*_ is included in the supplementary Information as Fig. S1.† In all cases, the patterns correspond to cubic polycrystalline specimens, with the characteristic reflections of skutterudite-type phases and with Sb as very minor impurity.

**Fig. 2:**
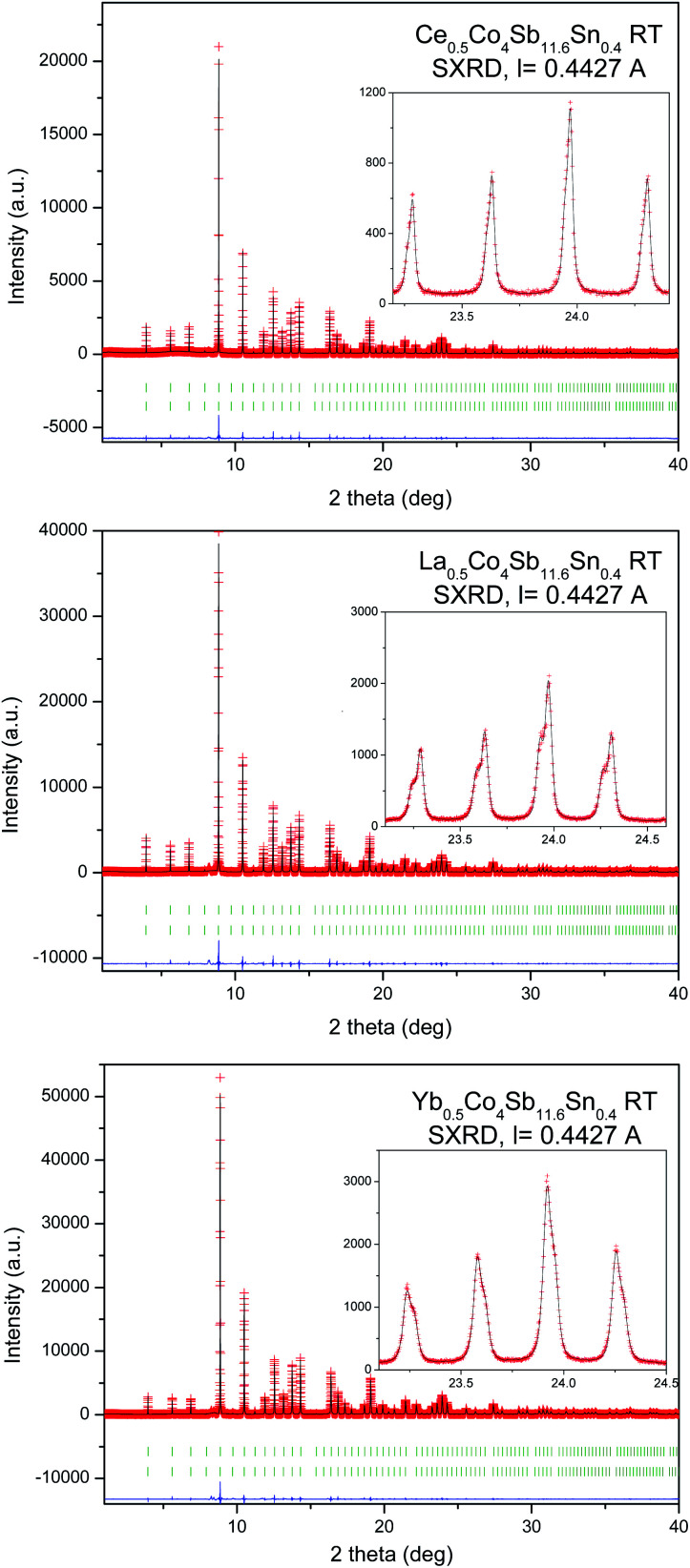
Observed (red crosses), calculated (black full line) and their difference (blue line), SXRD profiles for M_0.5_Co_4_Sb_11.6_Sn_0.4_ (M = Ce, La, Yb) at 295 K, with the peak splitting highlighted in the Insets. The two series of Bragg reflections correspond to the two coexistent skutterudite phases.

A SXRD study was essential to determine fine structural features of the synthesized R_0.5_Co_4_Sb_12_ skutterudites, which may help to understand the modifications of the transport properties. The crystal structure model was defined in the *Im*3̄ (no 204) space group. Co atoms are located at 8*c* (1/4, 1/4, 1/4), Sb and Sn atoms are distributed statistically over the 24*g* (0, *y*, *z*) sites, while the filler R atoms occupy the cages at 2*a* (0, 0, 0) Wyckoff positions. The diffraction peaks systematically show a splitting, especially noticeable at high scattering angles (Fig. 2, see insets), except for Ce_0.5_Co_4_Sb_11_Sn, which could be refined with a single skutterudite phase, since the SXRD pattern shows well-defined single reflections. This splitting is not noticeable in the laboratory XRD patterns, only in the SXRD diagrams with very high angular resolution. It is accounted for by admitting the presence of two different skutterudite phases, for which conspicuous differences in unit-cell parameters and filling fraction are found. Table 1 lists the main refined parameters, for the M_0.5_Co_4_Sb_11.6_Sn_0.4_ series (M = La, Ce, Yb), including occupation factors of the filler atoms, independently refined for each phase, unit-cell parameters, atomic positions, phase ratio, bond distances and agreement factors. The corresponding parameters for the Ce_0.5_Co_4_Sb_12−*y*_Sn_*y*_ series (*y* = 0.2, 1.0) are included in Table S1, in the ESI.† For the La-filled material with nominal composition La_0.5_Co_4_Sb_11.6_Sn_0.4_ there is a majority phase (66.4% in wt) with a lower refined filling fraction of *x* = 0.05, with a lattice parameter *a* = 9.04438(4) Å; whereas the minority phase (33.6%) has a superior filling fraction of *x* = 0.34, and a correspondingly larger lattice parameter of 9.06137(5) Å. A similar behavior is observed in the Ce specimens (Tables 1 and S2†), also consisting of a majority phase with a weak filling fraction (*x* = 0.085(7) for Sn_0.4_ and 0.063(3) for Sn_0.2_) and a minority phase (27.41% wt for Sn_0.4_ and 22.5% wt for Sn_0.2_) with *x* = 0.23(2), 0.179, respectively, and correspondingly large unit-cell sizes. The Ce_0.5_Co_4_Sb_11_Sn_1_ material displays a single skutterudite phase, with an actual Ce filling fraction of *x* = 0.157(7) and *a* = 9.04933(2) Å (Table S1†). Finally, the Yb material shows a distinct phenomenology in the sense that the minority phase is the one with lower filling fraction and lattice parameter; Fig. 2 shows that, in this case, the shoulder of the reflections appears at the high-angle side. Anyhow, it is remarkable that the filling fraction for the majority Yb phase in this work (*x* = 0.396(4)) is the highest observed, as reported in previous studies of HP-synthesized specimens.^29^ As described below, the transport properties of these compounds are influenced by the segregation into two differently-filled phases. Additionally, we have performed a temperature-dependent SXRD analysis of all the samples. The phase segregation remains stable up to 873 K, whereas the last collected pattern at 1073 K already shows a partial decomposition of the skutterudite phases. Fig. S2–S4† show the SXRD patterns at different temperatures (ESI†).

**Table 1:** Refined structural parameters of M_*x*_Co_4_Sb_12_ (M = Yb, Ce) at room temperature from SXRD data. Space group: Im3̄^a^

Nominal composition	La_0.5_Co_4_Sb_11.6_Sn_0.4_		Ce_0.5_Co_4_Sb_11.6_Sn_0.4_		Yb_0.5_Co_4_Sb_11.6_Sn_0.4_	
Refined composition	La_0.05_Co_4_Sb_11.6_Sn_0.4_	La_0.33_Co_4_Sb_11.6_Sn_0.4_	Ce_0.09_Co_4_Sb_11.6_Sn_0.4_	Ce_0.23_Co_4_Sb_11.6_Sn_0.4_	Yb_0.06_Co_4_Sb_11.6_Sn_0.4_	Yb_0.40_Co_4_Sb_11.6_Sn_0.4_
Phase abundance (%)	66.4(2)	33.6(2)	71.98(3)	28.02(1)	33.3(2)	66.7(3)
Lattice parameter (Å)	9.04439(4)	9.06138(5)	9.04483(4)	9.05343(6)	9.05007(5)	9.06408(4)
Volume (Å^3^)	739.840(5)	744.017(7)	739.949(5)	742.061(8)	741.235(8)	744.683(6)
*U*_11_ (Co)/Å^2*^	0.0086(3)	—	0.0082(4)	—	0.0024(2)	—
*U*_12_ (Co)/Å^2**^	−0.0007(5)	—	0.0013(7)	—	0.0004(4)	—
*y* (Sb)	0.33510(7)	0.3360(1)	0.3351(1)	0.3356(3)	0.3351(1)	0.33603(8)
*z* (Sb)	0.15812(7)	0.1593(1)	0.1579(1)	0.1592(3)	0.1577(1)	0.15911(8)
Occ. R (<1)	0.0525(4)	0.33(2)	0.087(6)	0.23(2)	0.062(5)	0.396(4)
*U*_11_ (Sb)/Å^2***^	0.0099(3)	—	0.0089(4)	—	0.0052(3)	—
*U*_22_ (Sb)/Å^2^	0.0152(4)	—	0.0133(6)	—	0.0098(4)	—
*U*_33_ (Sb)/Å^2^	0.0119(4)	—	0.0123(5)	—	0.0055(3)	—
*U*_23_ (Sb)/Å^2^	0.0006(3)	—	0.0011(4)	—	0.0008(2)	—
*U*_11_ (R)/Å^2****^	0.022(3)	—	0.034(7)	—	0.024(1)	—
d Co–Sb (Å)	2.5289(3)	2.5326(6)	2.5296(5)	2.530(1)	2.5319(5)	2.5341(3)
d_1_ Sb–Sb (Å)	2.860(1)	2.887(2)	2.857(2)	2.883(4)	2.854(2)	2.884(1)
d_2_ Sb–Sb (Å)	2.983(1)	2.973(2)	2.984(2)	2.977(4)	2.985(2)	2.973(1)
*R*_p_ (%)	6.75	—	7.28	—	5.25	—
*R*_wp_ (%)	8.56	—	9.64	—	7.50	—
*R*_exp_ (%)	4.80	—	7.01	—	4.21	—
*R*_Bragg_ (%)	2.33	2.32	3.56	7.14	2.23	1.45
*χ* ^2^(%)	3.17	—	1.89	—	3.18	—

a Sb at 24*g*, (0,*y*,*z*); Co at 8*c* (¼,¼,¼); R at 2a (0,0,0), Anisotropic U, Co:*U_11_ = *U*_22_ = *U*_33_; **U_12_ = *U*_23_ = *U*_13_; Sb:***U_12_ = *U*_13_ = 0; R:****U_11_ = *U*_22_ = *U*_33_.

We do not consider the phase segregation as impurities, since the distribution of the filler in two phases with different filling factor is a thermodynamic effect due to the inhomogeneous distribution of pressure within the Nb capsules, maximized in regions with grain-to-grain contacts. This useful effect for reducing the thermal conductivity is almost systematically present in our high-pressure preparations. However, the boundaries between segregated phases do act as scattering centers of both electrons and phonons.

### Atomic displacement factors

**Fig. 3:**
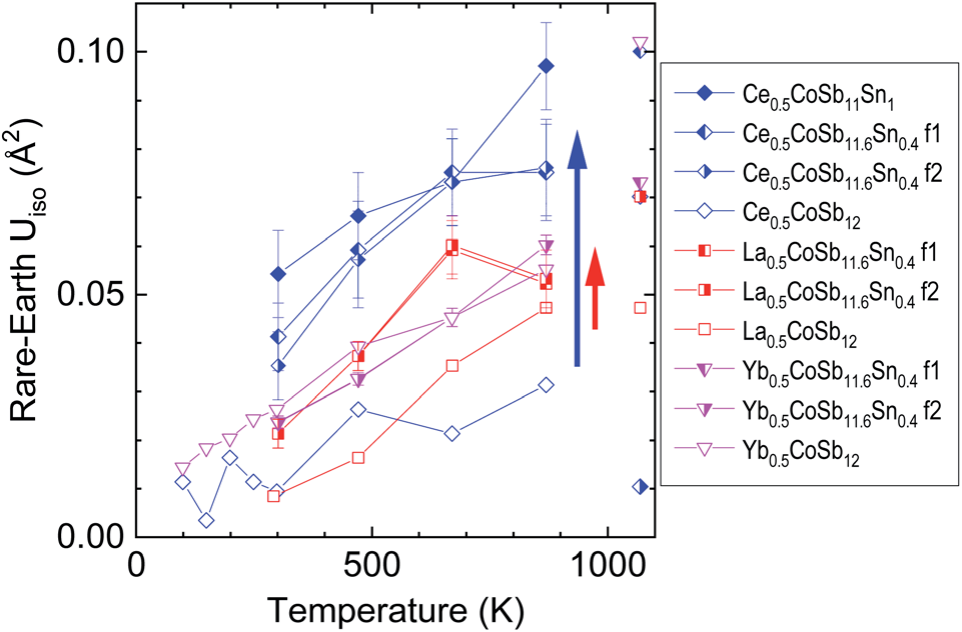
Atomic displacement factors (*U*_iso_) of the rare-earth fillers M_0.5_Co_4_Sb_12−*y*_Sn_*y*_ with M = Ce (blue rhombuses and triangles), La (red squares) and Yb (magenta triangles) in various skutterudite phases. The data for samples without Sn (*y* = 0, empty symbols) are reproduced from ref. 29 and 30. F1/f2 refers to the phase with higher/lower concentration of filler. The data points with partial decomposition at 1073 K are not connected to the rest.

The role of the rattlers (La, Ce, Yb) occupying the large 2*a* cages of the cubic structure is to contribute to a noteworthy reduction of the thermal conductivity due to the incoherent scattering of phonons derived from their rattling motion^29,30,34–37^. The evolution of the atomic displacement factors (ADP, converted to *U*_iso_) determined from the structural refinement of the SXRD data may give an interesting insight to the rattling motion of the fillers. Fig. 3 shows the temperature dependence of the *U*_iso_ values of the rare-earth fillers for the majority and minority phases of nominally half-filled M_0.5_CoSb_12−*y*_Sn_*y*_ with filling fraction fluctuation (M = La, Yb or Ce and *y* = 0.4) and for the monophase Ce_0.5_CoSb_11_Sn_1.0_, also including those without Sn substitution (*y* = 0).^29,30^ The *U*_iso_ magnitude increases with temperature approximately linearly between room temperature and 873 K, as expected for fillers in skutterudites, as in a typical Einstein-oscillator model. The slope is very similar for each phase, independent of filler-type and amount, or Sn-content. The crucial difference between the various phases is the overall constant offset of the *U*_iso_(T). This is usually called the disorder term and was associated with the expected rattling effect:^38^ the larger the filler-atom *U*_iso_, the stronger its effect on the lattice thermal conductivity.

Fig. 3 shows similar *U*_iso_ for the three Sn-free compounds, increasing from approx. 0.01 A^2^ at room temperature to approx. 0.04 A^2^ at 873 K for the Ce and La filled compounds and from 0.02 to 0.05 A^2^ for the Yb-filled one (data for Ce- and Yb-filled Sn-free samples were taken also below room temperature^30^). Whereas the filler-*U*_iso_ of the Sn-substituted Yb-filled compound is essentially unchanged, in both phases, the filler-*U*_iso_ of the Sn-substituted La-filled compound is larger by about 0.02 A^2^ (indicated by a red arrow). Remarkably, the largest increase due to Sn-substitution, about 0.04 A^2^ at 873 K (shown by a blue arrow) is observed for the Ce-filled compound. One of these compositions, Ce_0.5_Co_4_Sb_11.0_Sn_1.0_, is also the one that reaches the lowest lattice thermal conductivity of 1.0 W m^−1^ K^−1^, as discussed below. Such large *U*_iso_ might normally reflect compositional inhomogeneity, although the diffraction patterns of the Ce_0.5_Co_4_Sb_11.0_Sn_1.0_ compound could not be successfully refined as two phases of different Ce-compositions, unlike the other ones. The refined *U*_iso_ values have considerable uncertainty, but appear to indicate a larger increase with larger Sn-substitution. The *U*_iso_ values from diffractograms taken at the highest temperatures, 1073 K, are indicated but not connected to the rest, since they correspond to already partially thermally decomposed samples (see Fig. S3–S5†) and lack a consistent behaviour. To summarise, the largest increase in filler *U*_iso_ due to Sn-substitution is seen for Ce-filled compounds, whereas no increase is observed for the Yb-fillers in the Sn-substituted phase segregated samples, with the majority phase having higher filler fraction.

### Oftedal relation

**Fig. 4:**
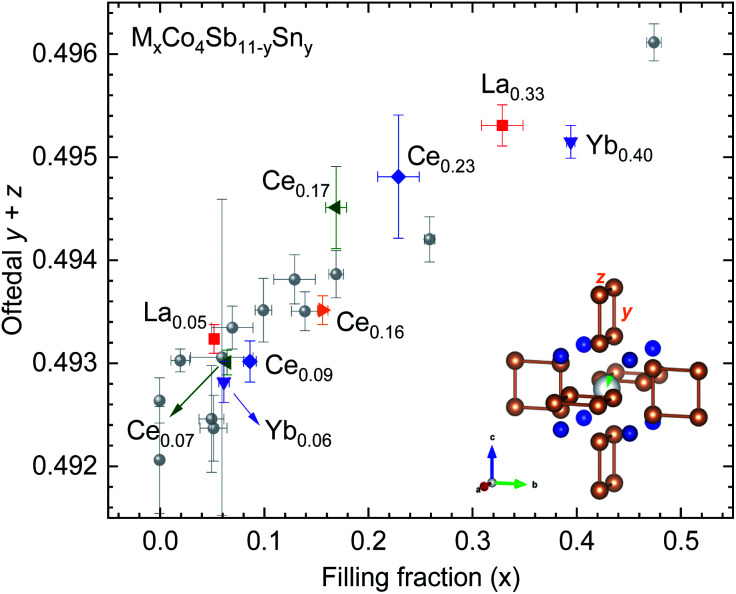
Sb (*y* + *z*) parameter *versus* filling fraction for some M_*x*_Co_4_Sb_11−*y*_Sn_*y*_ skutterudites. Data from several (filled and unfilled) undoped skutterudites prepared under high-pressure are extracted from previous work^28^ (grey circles). The inset highlights the skutterudite structure centered in the 2*a* position.

Fig. 4 shows the Sb structural parameters (*y* + *z*) *versus* the filling fraction for the filled and Sn-substituted skutterudites. We have imported previous results from Gainza *et al.*^28^ to compare these data to that of the Sn-free skutterudites. At a first glance, we can observe that our Sn-doped skutterudites follow the Oftedal relation: for increased filling fraction, the rectangular [Sb_4_] ring becomes closer to a square shape (perfect square means *y* + *z* = 0.5).

The maximum filling fraction shows the same trend on the rare-earth filler type with and without Sn doping: the Yb-filled composition has the highest filling fraction, followed by the La- and Ce-filled ones. The (*y* + *z*) parameter is similarly large for La- and Ce-filled as for Yb-filled CoSb_3_, although the latter has significantly higher filling fraction, highlighting the chemical differences between the filler elements. Remarkably, the filling fraction is increased by Sn-doping for Ce-, La- and Yb-filled skutterudites. Thus, the compounds display a lower structural distortion (more perfect (Sb, Sn)_4_ squares) than their undoped counterparts, which is beneficial for the thermoelectric performance because of the associated band convergence^28,39^ and enhanced resonant scattering of phonons.^40^

### Thermoelectric properties

**Fig. 5:**
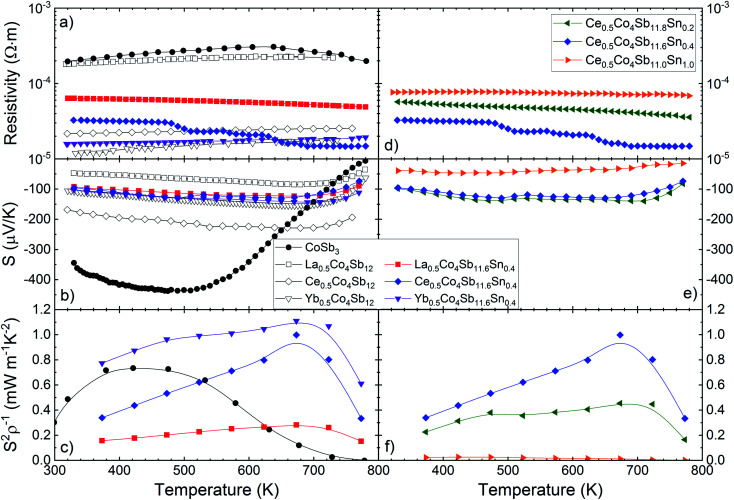
Temperature dependence of the (a) and (d) electrical resistivity, (b) and (e) Seebeck coefficient and (c) and (f) power factor (*S*^2^/*ρ*) of M_0.5_Co_4_Sb_12−*y*_Sn_*y*_ (M = La, Ce and Yb) (*y* = 0.2, 0.4, 1.0). Additional data are extracted from literature^27,29,30^ for the sake of comparison.

The electrical transport properties of the three filled skutterudites M_0.5_Co_4_Sb_11.6_Sn_0.4_ (M = La, Ce and Yb) are displayed in Fig. 5. The data of pristine CoSb_3−*δ*_ and some filled skutterudites (without Sn doping) prepared under the same high-pressure conditions^27,29,30^ are shown for the sake of comparison. Regardless of the filler element, the resistivity is significantly lower for filled skutterudites than for pure CoSb_3_, as shown in Fig. 5a and d. The resistivity of La- and Ce-filled skutterudites are almost constant from RT up to 800 K, but the Yb-filled one shows a more noticeable reduction, reaching a resistivity of 1.48 × 10^−5^ Ω m at 780 K, which is in agreement with other Yb-filled skutterudites doped with similar tin quantities.^25^ Sn-doping does not seem to affect significantly the resistivity, except for the La-filled skutterudite with a strong reduction from the undoped sample.

The Seebeck coefficient is plotted in the middle panels of Fig. 5(b and e). In this case, all the Seebeck coefficients are quite similar to each other, although they are significantly lower than the Seebeck coefficient of the unfilled CoSb_3_ compound below ∼680 K. Surprisingly, the Seebeck coefficient of the Sn-doped Yb-filled skutterudite is almost identical to its undoped counterpart, but the other ones differ in absolute value, although their behavior remains the same, with the bipolar conduction effect causing a reduction at high temperature.

Variations of the carrier density can be gleaned from the Seebeck coefficient values using the Single Parabolic Band approximation.^41^ For instance, the Seebeck coefficient of the Yb-filled compound remains practically unchanged with Sn substitution, an indication that the carrier density has not changed significantly either. On the contrary, the Seebeck coefficients of the Ce- and La- filled compounds do change when Sn is added into the structure, causing a rise or a reduction in its absolute value, respectively. For a carrier density estimation, see Table S2.†

In Fig. 5c and f, the power factor (*S*^2^/*ρ*) is displayed, calculated from experimental values of resistivity and Seebeck coefficient. The maximum of this power factor (∼1.1 mW m^−1^ K^−2^ at 673 K for the Yb_0.5_Co_4_Sb_11.6_Sn_0.4_ composition) is shifted to higher temperatures for Sn-doped filled skutterudites, compared with the unfilled pure CoSb_3_. The highest power factor, from 300 K up to 800 K, corresponds to Yb_0.5_Co_4_Sb_11.6_Sn_0.4_, although below other values reported elsewhere,^29–31^ but still over ∼0.7 mW m^−1^ K^−2^, until dramatically dropping above 700 K.

We can calculate the weighted mobility *μ*_w_ from the experimental data of electrical conductivity and Seebeck coefficient.^42^ In the free electron model, the weighted mobility is a property largely independent of doping, whereas the drift mobility varies with the carrier density. These two magnitudes are related through:1
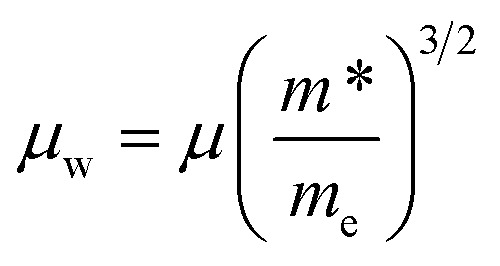


The main advantage of the weighted mobility is that it gives nearly the same information as the Hall mobility,^43^ so it can be used to analyze the charge carrier transport mechanism even when the information about carrier density is lacking.

**Fig. 6:**
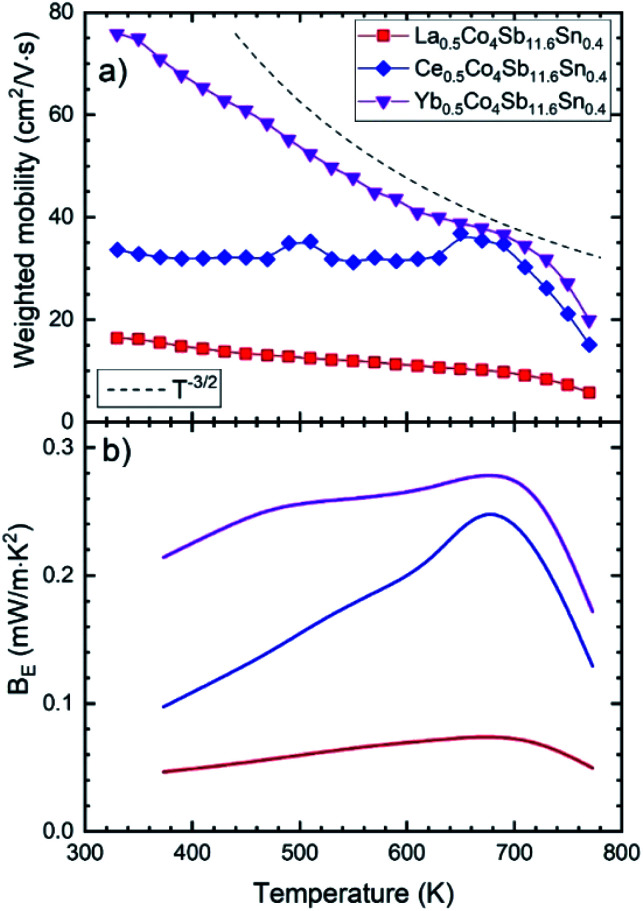
(a) Weighted mobility calculated from experimental Seebeck coefficient and resistivity. It decreases with temperature, as expected from the phonon scattering of electrons. (b) Temperature dependent electronic quality factor (*B*_E_).

For most good thermoelectric materials, this weighted mobility decreases continually due to the phonon scattering effect.^42^ This happens also in each of our Sn-doped filled compounds (Fig. 6). The *μ*_w_ decreases monotonically as temperature increases, and after the onset of bipolar conductivity around 700 K, the slope of this reduction becomes greater. In the simple model for acoustic phonon scattering, this decrease with temperature follows a *T*^−3/2^ relation, which is clearly seen in the Yb-filled compound.

The expected quality of different thermoelectric compounds can be compared with pairs of electrical resistivity and Seebeck coefficient data, by defining the electronic quality factor (*B*_E_).^44^ Furthermore, this parameter is temperature independent for reasonably good thermoelectric materials, so any variation of *B*_E_ with temperature will reveal unusual effects such as non-parabolic bands, band convergence, different scattering mechanisms or bipolar conduction.

The bigger *B*_E_ for the Ce- and Yb-filled compounds indicates the effect of band convergence for both compositions, with the almost temperature independent flat line for Yb-the result that we are looking for, since band converge has been proven useful to enhance the thermoelectric performance in skutterudites.^8,28,39^ This band convergence is also the reason why the electronic quality factor increases somewhat with temperature, until the bipolar conduction kicks in.

**Fig. 7:**
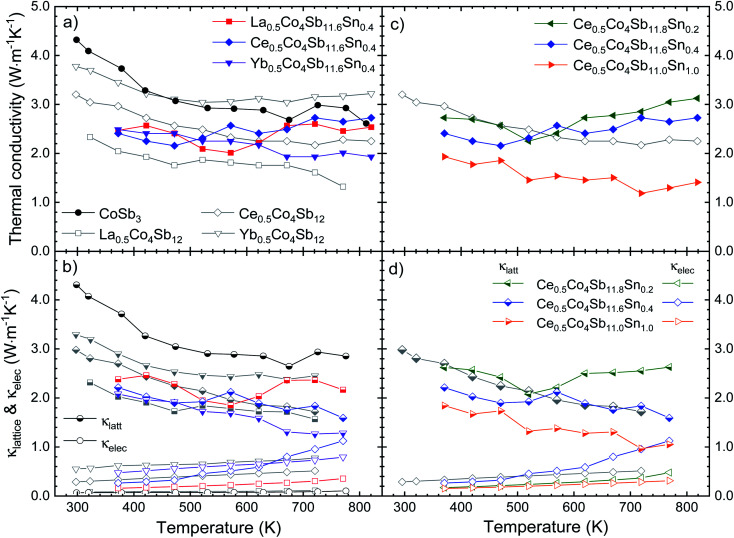
(a) and (c) Total (*κ*_tot_) and (b) and (d) electronic (*κ*_elec_) and lattice (*κ*_lattice_) thermal conductivity as a function of temperature.

The temperature dependent thermal conductivity is plotted in Fig. 7. The lattice thermal conductivity is obtained by subtracting the electronic contribution from the *κ*_tot_, calculated from the resistivity using the Wiedemann–Franz law, *κ*_e_ = *LT*/*ρ*. The L parameter is the Lorenz number, calculated from the single parabolic band model.^41^

Near room temperature, around 360 K, the thermal conductivity for the Sn-doped skutterudites is very similar, ∼2.5 W m^−1^ K^−1^, significantly lower than that of high pressure synthesized CoSb_3_ at the same temperature, 3.3 W m^−1^ K^−1^ (already reduced by ∼50% compared to conventional CoSb_3_). The La- and Ce-filled compositions show an increase in the *κ*_tot_ beyond 550 K, reaching that of the pure compound, around 2.5–2.7 W m^−1^ K^−1^. This increment has been reported before,^22^ with even higher conductivities,^20,25,45^ ∼3 W m^−1^ K^−1^. In the case of the Ce-doped compound, this relatively high total thermal conductivity is dominated by the electronic part due to its low resistivity at high temperature (Fig. 5a), also observed as the *κ*_e_ (Fig. 7b) approaching the value of *κ*_latt_ at 773 K. In the La-filled compound, the high lattice thermal conductivity also contributes to its relatively high *κ*_tot_. For Yb_0.5_Co_4_Sb_11.6_Sn_0.4_, the total thermal conductivity as well as the lattice conductivity are the lowest of the series, which may be related to the superior filling fraction of the Yb filler, observed for the majority skutterudite phase (*x* = 0.39, Table 1). This total thermal conductivity of 1.9 W m^−1^ K^−1^ at 823 K is lower than that reported in other Yb single-filled skutterudites, such as that for Yb_0.35_Co_3.75_Fe_0.25_Sb_12_ which is 2.7 W m^−1^ K^−1^,^46^ or for the Yb_0.30_Co_4_Fe_0.25_Sb_11.8_Sn_0.2_ compound, with a total thermal conductivity of 3.0 W m^−1^ K^−1^.^25^ Sn-doping causes a remarkable decrease of the lattice thermal conductivity in the Yb-filled skutterudite, contrary to the Ce- and La-filled compounds. Another interesting trend is that observed in the Ce_0.5_Co_4_Sb_12−*y*_Sn_*y*_ (*y* = 0.2, 0.4, 1.0) compounds, where the thermal conductivity progressively decreases as the Sn doping level increases (Fig. 7). The Ce_0.5_Co_4_Sb_11.6_Sn_0.4_ compound shows a *κ*_latt_ around 2.2 W m^−1^ K^−1^ at room temperature, which is in agreement with the general trend expected for these filling fraction values (of ∼0.23, see Table 1) in rare-earth-filled skutterudites.^14^ The end member Ce_0.5_Co_4_Sb_11_Sn_1.0_ is the material with the lowest *κ*_tot_ and *κ*_latt_, indicating that the effect of disorder at the (Sb, Sn) positions (24*g* sites of the *Im*3̄ space group) predominates over the absence of phase segregation. In comparison with the Ce-filled skutterudites synthesized following a solubility design,^14^ showing a thermal conductivity of 2.8 W m^−1^ K^−1^, we achieve a higher reduction in this *κ*_tot_.

This disorder effect, or point defect scattering, is caused by the replacement of Sn in the position of the Sb atom. These point defects cause lattice distortion, so the periodic potential field around that site deviates from the normal arrangement. Therefore, these point defects become scattering centers for electrons or phonons.^47^ This phenomenon related to the dopant atoms can explain the reduction in the thermal conductivity with increasing tin quantity, as is shown in Fig. 7c.

**Fig. 8:**
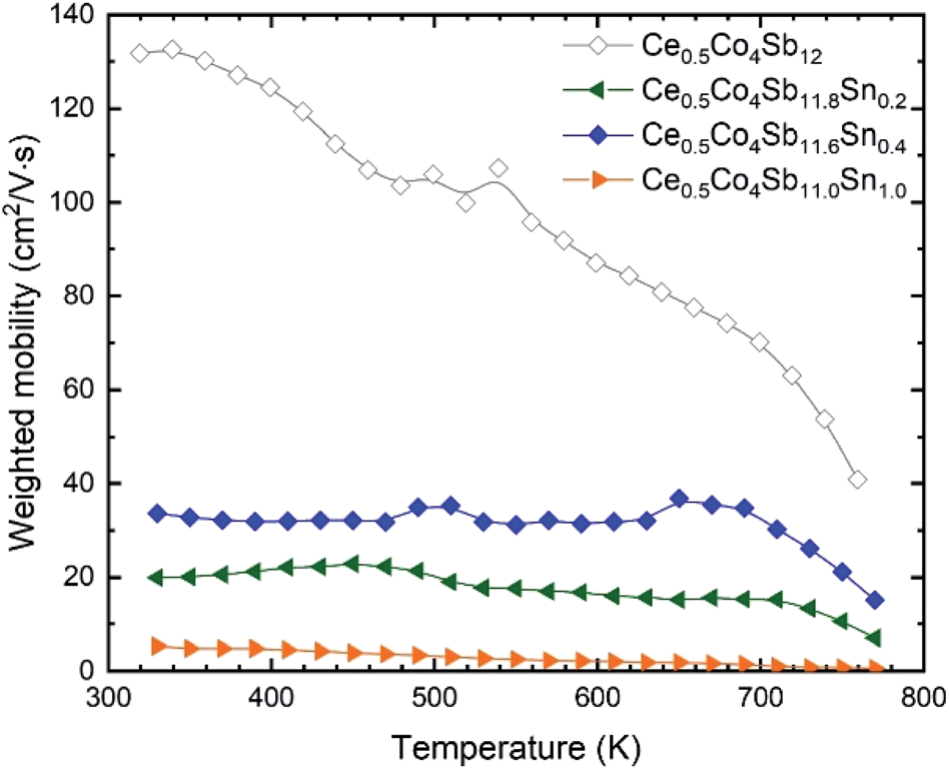
Weighted mobility of Ce_0.5_Co_4_Sb_12−*y*_Sn_*y*_. Among the doped compositions, the Ce_0.5_Co_4_Sb_11.6_Sn_0.4_ shows the best weighted mobility from room temperature up to 800 K.

Sn doping decreases the weighted mobility, as shown in Fig. 8 for the Ce_0.5_Co_4_Sb_12−*y*_Sn_*y*_ (*y* = 0, 0.2, 0.4, 1.0) series. Near room temperature, the difference is large but since the weighted mobility of the undoped compound decreases rapidly, above 700 K it is less pronounced. The weighted mobility of Ce_0.5_Co_4_Sb_12_ is 40.89 cm^2^ V^−1^ s^−1^ at 760 K, a value comparable to that of the Ce_0.5_Co_4_Sb_11.6_Sn_0.4_ below 700 K. The weighted mobility of Ce_0.5_Co_4_Sb_12_ shows the typical relation with temperature for acoustic phonon scattering, along with the decrease above 700 K due to bipolar conduction. Point defect scattering due to Sn dopants decreases both the lattice thermal conductivity (beneficial for a thermoelectric) and the weighted mobility (detrimental).

## Discussion

Sn doping has rather disparate effects on the atomic displacement (*U*_iso_) of the filler, lattice thermal conductivity and electronic properties, depending on the type of filler. Sn doping increases modestly the *U*_iso_ of La, nevertheless it also increases the *κ*_lattice_. It increases *U*_iso_ of Ce dramatically and decreases somewhat *κ*_lattice_, whereas the *U*_iso_ of Yb remains unchanged with Sn doping yet the corresponding *κ*_lattice_ is greatly decreased. This is quite contrary to the expectation that lattice thermal conductivity reduction in filled skutterudites would be governed by the overall filler motion.^13^ On the other hand, the electronic power factor due to Sn-doping is greatly improved for La-filling, is somewhat decreased for Yb-filling and does not change consistently for Ce-filling.

We must take into account that unfilled CoSb_3_ is an intrinsic semiconductor with a carrier concentration below 10^18^ cm^−3^. To achieve a high power factor in filled skutterudites, a carrier concentration between 10^20^–10^21^ cm^−3^ is required.^14^ The filling fraction limit (FFL) in CoSb_3_ is reported to be 0.2 for ytterbium and 0.1 for cerium.^18^ There are already some reports for Yb-filled skutterudites reaching a filling fraction close to 0.2,^48^ or even above 0.25,^14^ but for the case of Ce, its filling fraction barely reaches a value of ∼0.15 following a solubility design.^14^ This low filling fraction and FFL for Ce is the main limiting factor to achieve the optimum carrier concentration in Ce-filled skutterudites.

In the present work, our aim was to increase the low experimental filling fraction of Ce in CoSb_3_ skutterudites by means of Sn doping, approaching to that reported for ytterbium. We empirically obtain from SXRD data refinement a filling fraction of 0.23(2) for the Ce-filled compound in the majority phase, improving the previously reported data and paving the way to adjust more conveniently the carrier density in this type of materials.

The effective mass of most filled skutterudites (La-, Ce-, Yb-, Nd-, *etc.*)^13^ show an effective mass around 1.5 and 1.7 m_e_. Therefore, we have calculated the estimated carrier density for each composition in order to compare them properly, using the single parabolic band approximation^41^ at 330 K (Table S2†). As shown, the carrier density of the Yb-filled skutterudite remains practically unchanged even with Sn substitution, so this strategy on its own is not the best to enhance its thermoelectric performance, notwithstanding the strong reduction in thermal conductivity.

On the other hand, the carrier density reduction for the La- compound is readily apparent when we incorporate Sn into the structure. The increment observed in the Seebeck coefficient is due to this effect.

Finally, for the Ce-filled compound we observe an evident increase in the carrier density. This is the south after characteristic, since in skutterudites a carrier density above 10^20^ cm^−3^ is needed for a high power factor.

Since the power factor depends heavily on the electronic doping level, a better measure of the electronic changes is the *B*_E_ (Fig. 6b) quality factor, which is consistently improved for Yb-filling due to Sn-doping. These differences may be understood considering each of the filler elements separately, and the effects of simultaneously doping and filling the skutterudite structure, along with the chemical nature of the rare-earth cation. In the case of La-filled CoSb_3_, electrical and thermal transport are dominated by the more conductive behavior of the R-rich and R-poor phases, respectively. Sn-doping increases the filling fraction of the La-rich phase from La_0.17_ to La_0.34_, yielding a higher carrier concentration and optimizing the relation of electrical conductivity and Seebeck coefficient, and thus, increasing the power factor. However, compared to the undoped sample, the ratio of La-poor phase is much higher. This would increase the lattice thermal conductivity, as the filling fraction is a crucial factor determining phonon scattering.^14,29,49^ Ce-filled CoSb_3_ has been typically described showing Ce^4+^ valence state^50^ (in contrast to trivalent La^3+^ ions), which could explain the significant increase of *U*_iso_: upon Sn doping, the Sb/Sn sublattice would display charge imbalance due to the acceptor character of the Sn atoms. According to Fajan's rules,^51^ smaller size and higher positive charge of cations makes them more polarizing and able to deform electronic clouds, which would agree with local Ce^4+^ shifts regarding the canonic crystallographic position, according to the presence of Sn in their surroundings. In spite of this disorder within the crystal structure, the lattice thermal conductivity would show a slight reduction only due to the high ratio of Ce-poor phase (70%). The Yb-filled CoSb_3_ compounds display a larger filling fraction limit, due to the stability of the Yb^2+^ ion.^18^ We can expect that the charge imbalance produced by Sn in the Sb sublattice can be compensated by partial oxidation to Yb^3+^, and therefore, a stable *U*_iso_ regarding Sn doping may be expected. Furthermore, this compensation also allows for a similar Fermi level and power factor of the undoped and doped samples. At the same time, the filling fraction is increased to Yb_0.40_ in the predominant Yb-rich phase, which along with the disorder induced by Sn doping, strongly enhances phonon scattering, yielding a significant reduction of the lattice thermal conductivity. Besides, phase segregation was not observed in undoped Yb-filled samples,^30^ which will now further contribute as another phonon scattering mechanism. We may conclude that Sn-doping effectively reduces lattice thermal conductivity in Yb-filled CoSb_3_ by allowing an increased filling fraction while retaining good electrical properties, yielding an overall improvement of the thermoelectric performance. While this is not the case for Ce-and La-filled compounds, it can be directly related to the physical and chemical differences of the rare-earth elements, and their interaction with the skutterudite system.

**Fig. 9:**
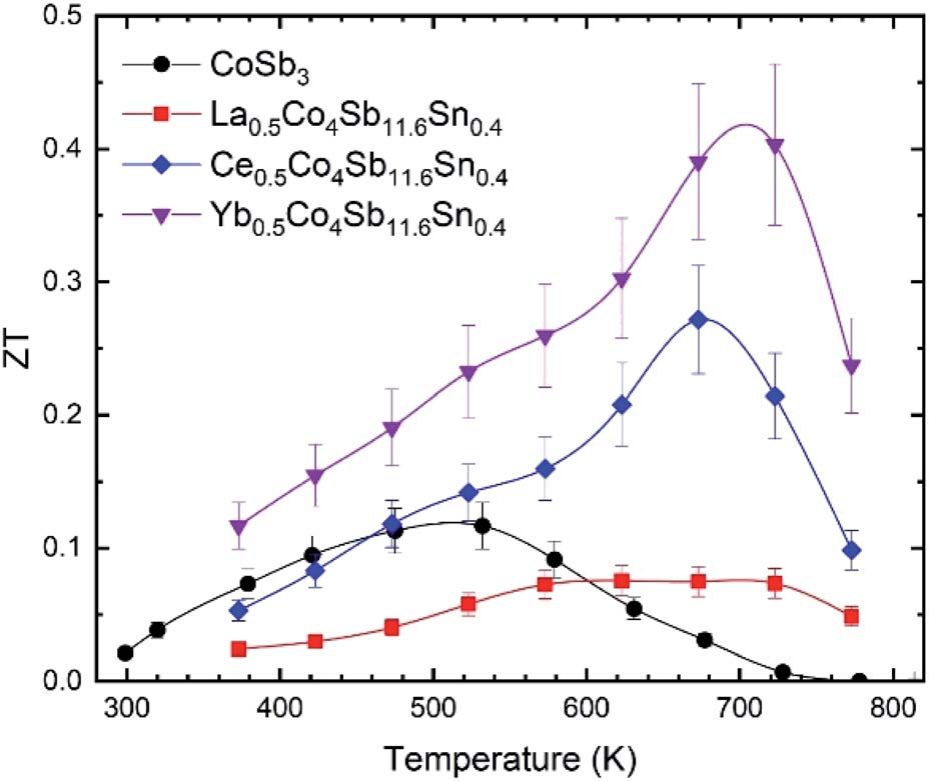
Temperature dependence of the figure of merit, ZT, of CoSb_3_, La_0.5_Co_4_Sb_11.6_Sn_0.4_, Ce_0.5_Co_4_Sb_11.6_Sn_0.4_ and Yb_0.5_Co_4_Sb_11.6_Sn_0.4_. The error bars are placed considering an error of ±15%.

The ZT figure of merit of pure CoSb_3_ and the three compositions of the M_0.5_Co_4_Sb_11.6_Sn_0.4_ series are displayed in Fig. 9. All of them have higher figure of merit at ∼700 K than the pure CoSb_3_. The one filled with La is worse than the unfilled compound at temperatures near RT, but it is still better than its undoped analog, La_0.5_Co_4_Sb_12_.^29^ The maximum figure of merit corresponds to the Yb_0.5_Co_4_Sb_11.6_Sn_0.4_ skutterudite, reaching ∼0.4 at ∼700 K, which is higher than the figure of merit reported for Yb_0.5_Co_4_Sb_12_ of 0.3.^30^

**Fig. 10:**
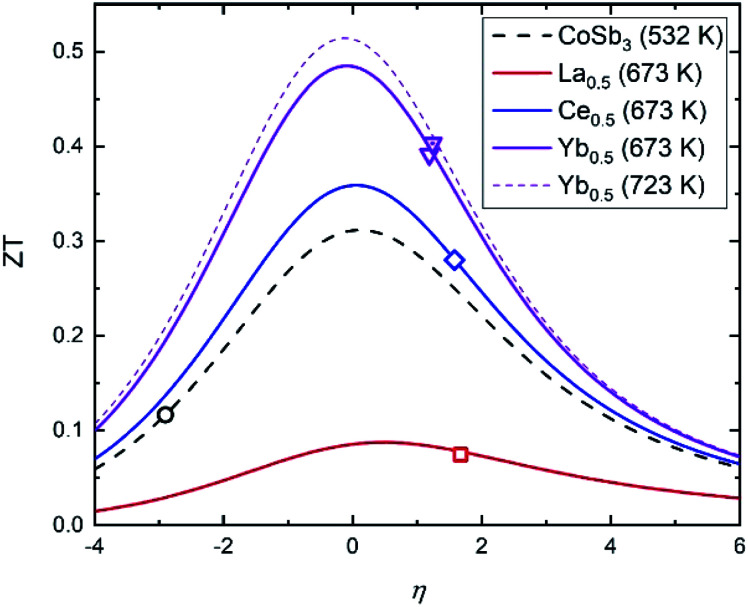
ZT *vs.* reduced chemical potential (*η*) curves predicted by the quality factor at 532 K (CoSb_3_), 673 K (La_0.5_Co_4_Sb_11.6_Sn_0.4_, Ce_0.5_Co_4_Sb_11.6_Sn_0.4_ and Yb_0.5_Co_4_Sb_11.6_Sn_0.4_) and 723 K (Yb_0.5_Co_4_Sb_11.6_Sn_0.4_). The points represent the experimental data.

This figure of merit can be analyzed as a function of two independent variables: the thermoelectric quality factor *B*, which indicates the maximum available ZT with proper charge carrier density based on band structure and lattice thermal conductivity, and the reduced chemical potential *η*.^52^ This reduced chemical potential depends on the chemical doping and the temperature, and can be extracted from the Seebeck coefficient.^41^ This parameter gives us information about the doping level, which can be altered by defects and impurities. Therefore, to optimize the ZT, this reduced chemical potential must be adjusted to the best value by doping the material correctly. Fig. 10 displays the ZT *vs. η* curves for several compositions at the temperatures where they reach their highest figures of merit. The reduced chemical potentials in all the filled skutterudites are quite different than that of the pure compound, but very similar to each other. The *η* parameter could be further optimized to reach higher figures of merit (up to 0.5 for the Yb_0.5_Co_4_Sb_11.6_Sn_0.4_ composition), but the improvement compared to pure CoSb_3_ is readily apparent and demonstrates the effect of the reduced lattice thermal conductivity and band convergence in addition to electronic doping.

## Conclusions

M_*x*_Co_4_Sb_12−*y*_Sn_*y*_ (M = La, Ce, Yb) filled skutterudites with Sn doping were successfully stabilized with a high-pressure synthesis. The SXRD patterns display conspicuously split peaks, interpreted as a phase segregation of twin skutterudite phases with distinct lattice parameters due to filling fraction fluctuation, except for Ce_0.5_Co_4_Sb_11.0_Sn_1.0_. The M filler atoms contribute to lower the thermal conductivity with their rattling motion and the filling fraction fluctuation, and also improve the electronic conductivity and the power factor through charge transfer. The filling fraction of the Ce-filled compound is increased to 0.23(2) in the majority phase, demonstrating the potency of Sn-substitution to increase the filler occupancy. The disorder induced by Sn doping at the Sb sublattice, maximized in Ce_0.5_Co_4_Sb_12−*y*_Sn_*y*_, further reduces *κ*_lattice_ and thus the total thermal conductivity. However, this disorder also lowers the weighted mobility. The combination of improved electrical conductivity and reduced thermal conductivity confer to Yb_0.5_Co_4_Sb_11.6_Sn_0.4_ an optimum ZT of 0.40. The electronic quality factor of this composition is nearly constant from 400 K up to 700 K, indicating the positive effect of band convergence.

## Conflicts of interest

There are no conflicts to declare.

## Supplementary Material

RA-011-D1RA04270J-s001
