# Supplementary material for: Strongly reduced lattice thermal conductivity in Sn-doped rare-earth (M) filled skutterudites MxCo4Sb12−ySny, promoted by Sb–Sn disordering and phase segregation
Source: RSC Adv. 2021 Aug 3;11(42):26421–31. doi: 10.1039/d1ra04270j (PMC9037351; doi:10.1039/d1ra04270j)
Supplement: RA-011-D1RA04270J-s001 [file RA-011-D1RA04270J-s001.pdf]

Electronic Supplementary Material (ESI) for RSC Advances.
This journal is © The Royal Society of Chemistry 2021

SUPPLEMENTARY INFORMATION

Strongly reduced lattice thermal conductivity in Sn-doped rare-earth (M) filled skutterudites $M_xCo_4Sb_{12-y}Sn_y$, promoted by Sb-Sn disordering and phase segregation

J. Gainza,[a,] F. Serrano-Sánchez,[a] N. M. Nemes,[a,b] O. J. Dura,[c] J.L. Martínez,[a] F. Fauth,[d] J.A. Alonso[a]*

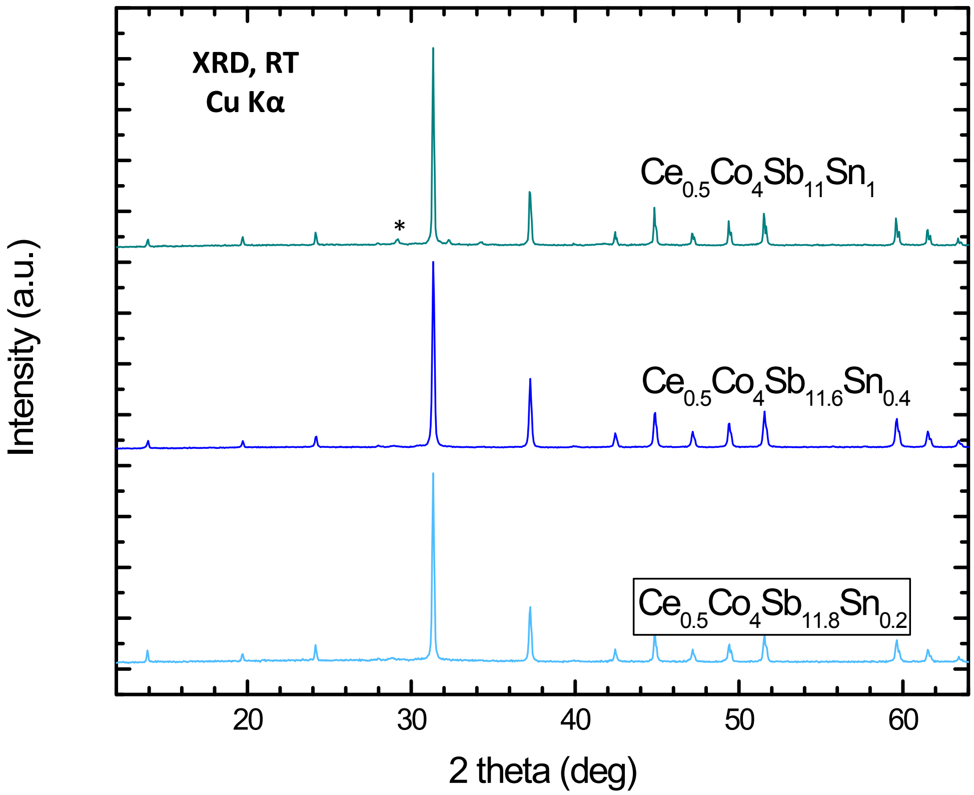

Figure S1. Laboratory XRD patterns of as-grown $Ce_{0.5}Co_4Sb_{12-y}Sb_y$ (y = 0.2, 0.4, 1.0) at room temperature. The star corresponds to the most intense reflection of Sb impurity.

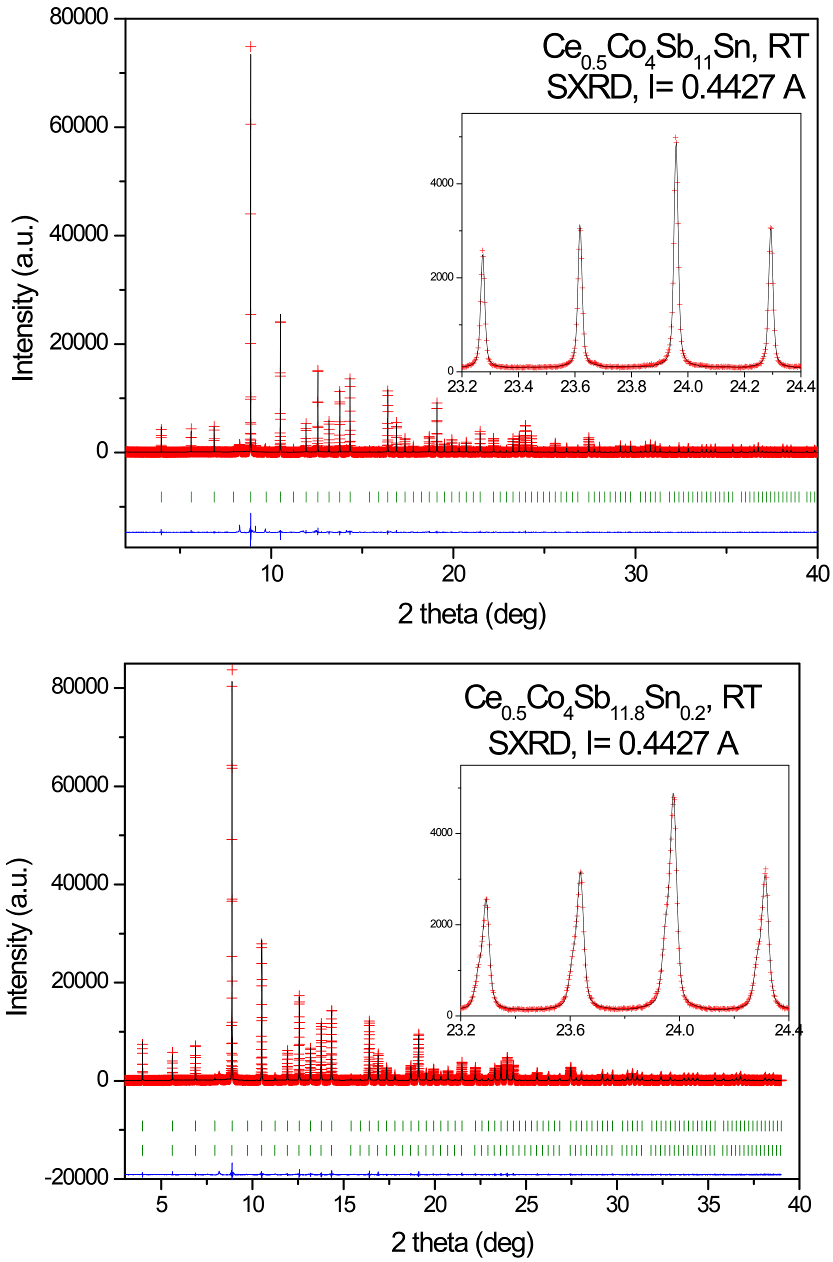

Figure S2. Observed (red crosses), calculated (black full line) and their difference (blue line), SXRD profiles for $Ce_{0.5}Co_4Sb_{12-y}Sn_y$ (y = 0.2, 1) at 295 K, with the peak splitting highlighted in the Insets. For x = 0.2, the two series of Bragg reflections correspond to the two coexistent skutterudite phases.

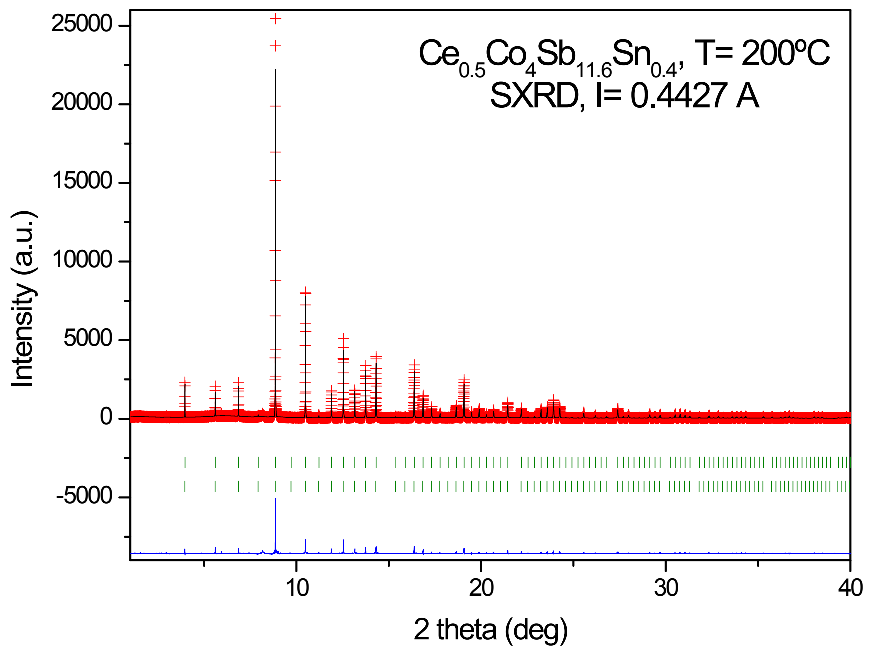
Ce0.5Co4Sb11.6Sn0.4, T= 200ºC
SXRD, l= 0.4427 A
Intensity (a.u.)
2 theta (deg)

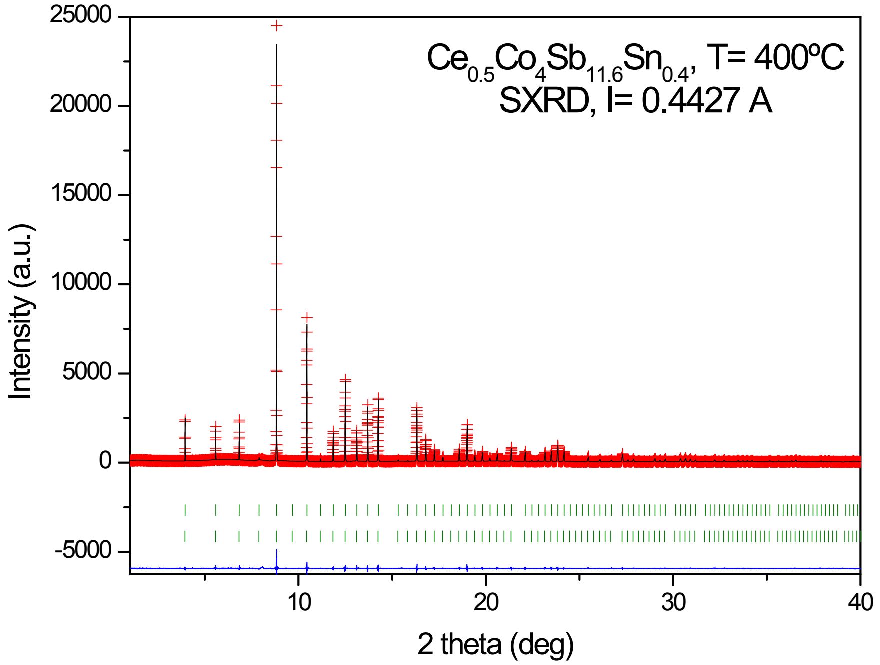
Ce0.5Co4Sb11.6Sn0.4, T= 400ºC
SXRD, l= 0.4427 A
Intensity (a.u.)
2 theta (deg)

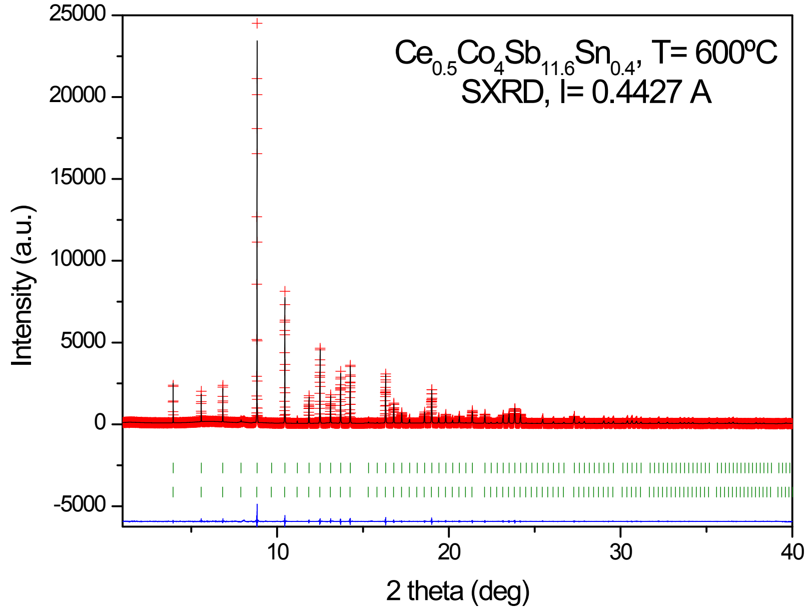

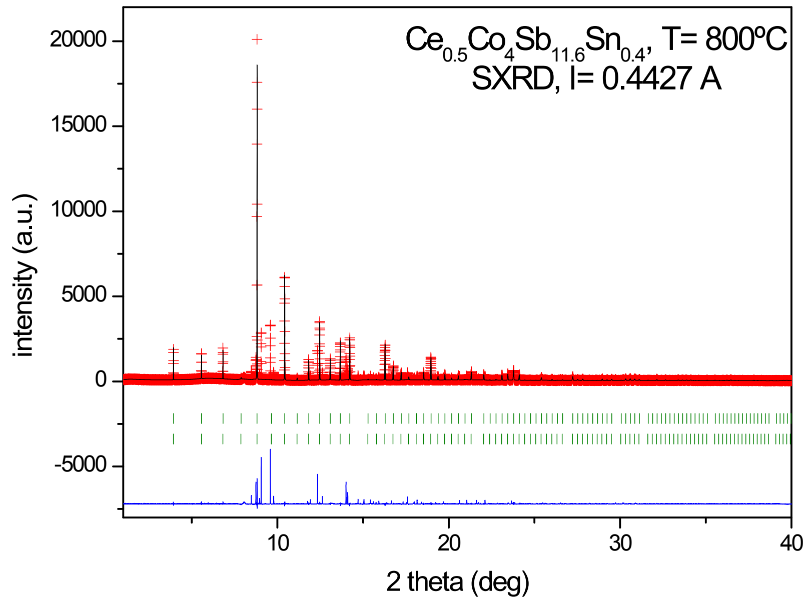

Figure S3. Observed (red crosses), calculated (black full line) and their difference (blue line), SXRD profiles for $Ce_{0.5}Co_4Sb_{11.6}Sn_{0.4}$ at 200, 400, 600 and 800ºC.The two series of Bragg reflections correspond to the two coexistent skutterudite phases. At 800ºC, the incipient decomposition of the skutterudite phase is observed.

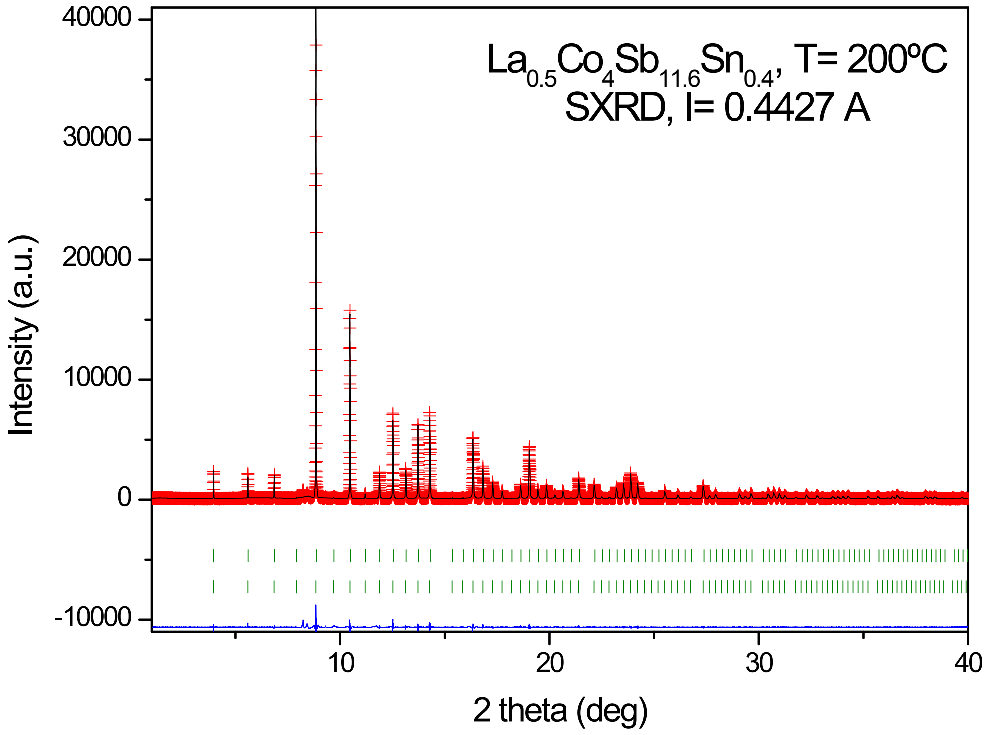
La0.5Co4Sb11.6Sn0.4, T= 200ºC
SXRD, l= 0.4427 A
Intensity (a.u.)
2 theta (deg)
40000
30000
20000
10000
0
-10000
10
20
30
40

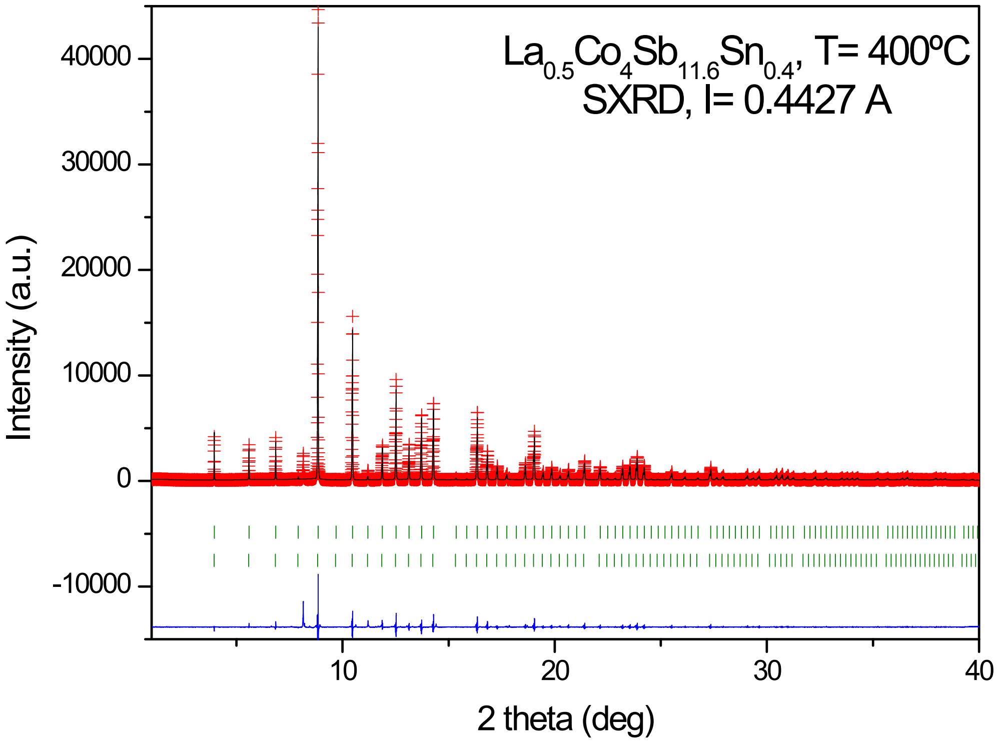
La0.5Co4Sb11.6Sn0.4, T= 400ºC
SXRD, l= 0.4427 A
Intensity (a.u.)
2 theta (deg)
40000
30000
20000
10000
0
-10000
10
20
30
40

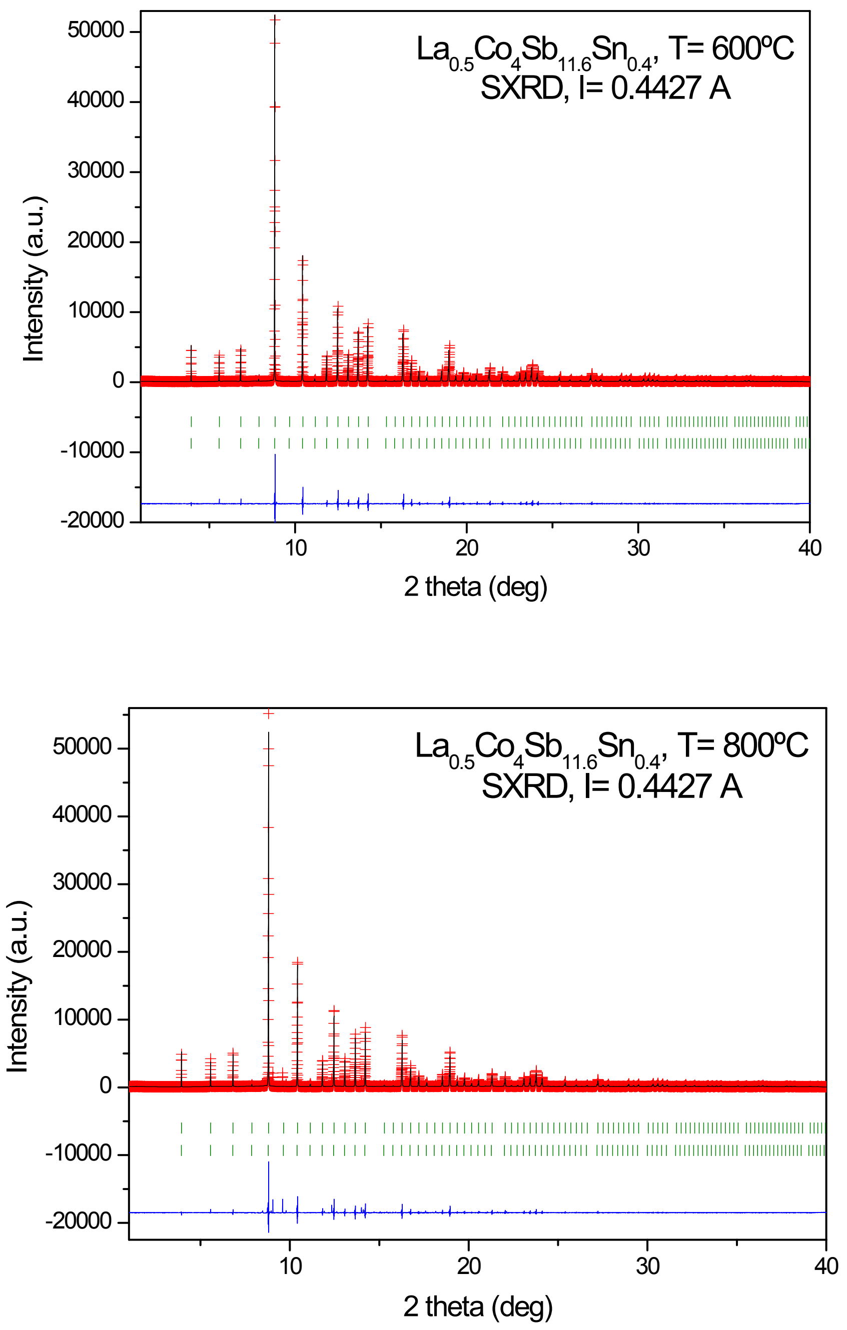

Figure S4. Observed (red crosses), calculated (black full line) and their difference (blue line), SXRD profiles for $La_{0.5}Co_4Sb_{11.6}Sn_{0.4}$ at 200, 400, 600 and 800ºC.The two series of Bragg reflections correspond to the two coexistent skutterudite phases. At 800ºC, the incipient decomposition of the skutterudite phase is observed.

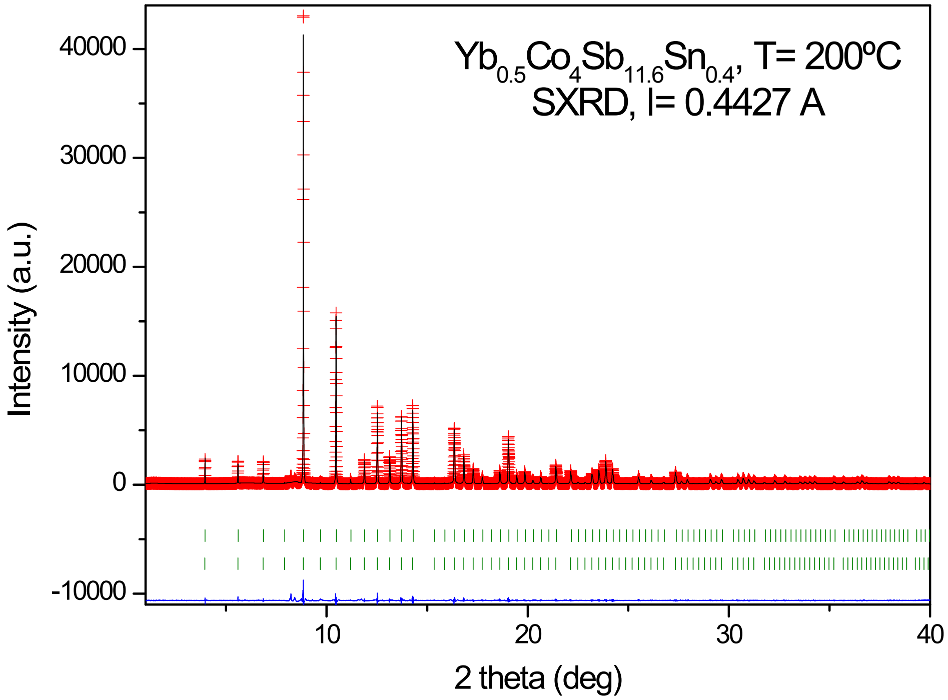

$Yb_{0.5}Co_4Sb_{11.6}Sn_{0.4}$, T= 200ºC
SXRD, l= 0.4427 A
Intensity (a.u.)
2 theta (deg)
40000
30000
20000
10000
0
-10000
10
20
30
40

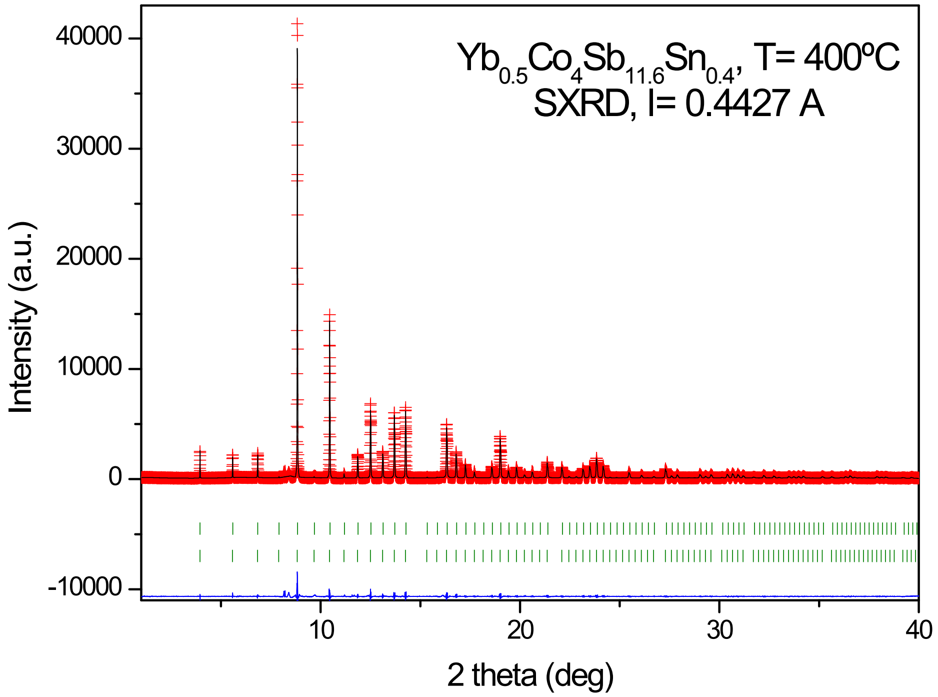

$Yb_{0.5}Co_4Sb_{11.6}Sn_{0.4}$, T= 400ºC
SXRD, l= 0.4427 A
Intensity (a.u.)
2 theta (deg)
40000
30000
20000
10000
0
-10000
10
20
30
40

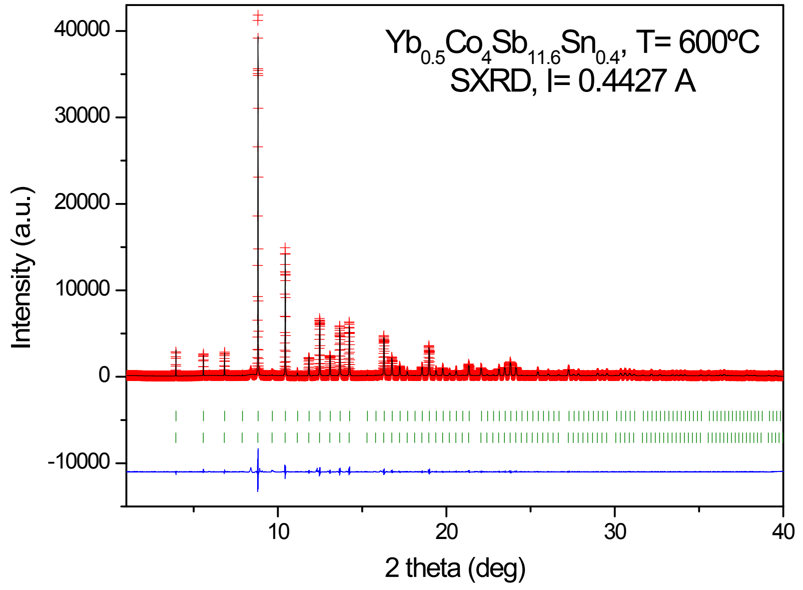

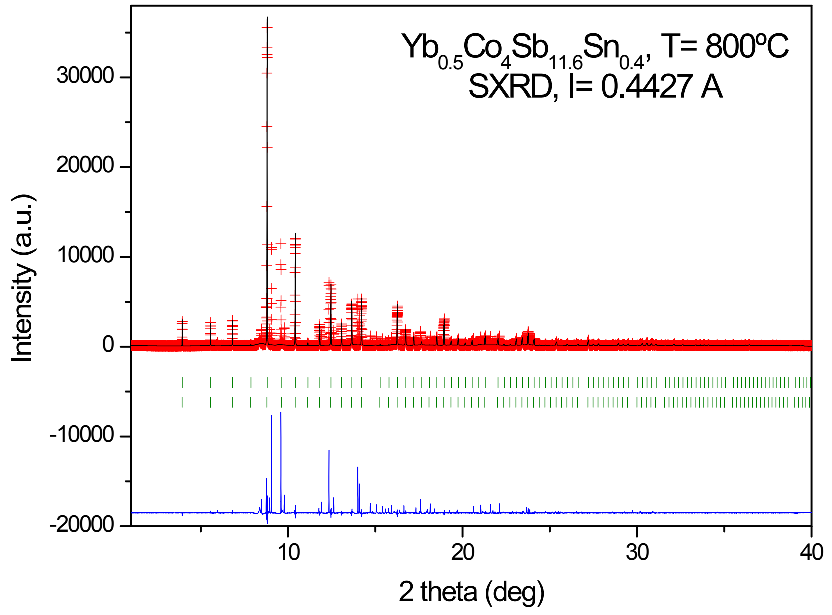

Figure S5. Observed (red crosses), calculated (black full line) and their difference (blue line), SXRD profiles for $Yb_{0.5}Co_4Sb_{11.6}Sn_{0.4}$ at 200, 400, 600 and 800ºC.The two series of Bragg reflections correspond to the two coexistent skutterudite phases. At 800ºC, the incipient decomposition of the skutterudite phase is observed.

Table S1. Refined structural parameters of $Ce_{0.5}Co_4Sb_{12-y}Sn_y$ (y = 0.2, 1.0) at room temperature from SXRD data. Space group: $Im\bar{3}$.

**Nominal Composition**	$Ce_{0.5}Co_4Sb_{11.8}Sn_{0.2}$		$Ce_{0.5}Co_4Sb_{11\text{-}0}Sn_{1.0}$
**Refined Composition**	$Ce_{0.07}Co_4Sb_{11.8}Sn_{0.2}$	$Ce_{0.17}Co_4Sb_{11.8}Sn_{0.2}$	$Ce_{0.16}Co_4Sb_{11.0}Sn_{1.0}$
**Phase abundance (%)**	77.6(3)	22.4(2)	100%
**Lattice parameter (å)**	9.04203(3)	9.05289(4)	9.04933(2)
**Volume (Å^3)**	739.260(4)	741.928(6)	741.05(1)
**U_{11} (Co) / Å^2** *	0.0052(3)		0.0050(3)
**U_{12} (Co) / Å^2** **	0.0000(4)		0.0004(5)
***y* (Sb)**	0.33506(6)	0.3354(2)	0.33523(7)
***z* (Sb)**	0.15793(6)	0.1591(2)	0.15827(7)
**Occ. R (<1)**	0.065(3)	0.17(1)	0.157(5)
**U_{11} (Sb) / Å^2** ***	0.0070(3)		0.0074(3)
**U_{22} (Sb) / Å^2**	0.0118(4)		0.0109(4)
**U_{33} (Sb) / Å^2**	0.0087(4)		0.0081(4)
**U_{23} (Sb) / Å^2**	0.0006(2)		0.0011(3)
**U_{11} (R) / Å^2** ****	0.031(6)		0.054(7)
**d Co-Sb (å)**	2.5287(3)	2.5292(7)	2.5302(3)
**d_1 Sb-Sb (å)**	2.8560(9)	2.881(2)	2.8645(9)
**d_2 Sb-Sb (å)**	2.9828(9)	2.980(2)	2.9821(9)
**R_p (%)**	5.98		10.584
**R_{wp} (%)**	7.67		18.623
**R_{exp} (%)**	4.08		5.942
**R_{Bragg} (%)**	1.36	2.99	1.937
**χ^2(%)**	3.54		4.15

Sb at 24*g*, (*0*,*y*,*z*); Co at 8*c* (¼,¼,¼); R at 2a (0,0,0)
Anisotropic U
Co: * $U_{11} = U_{22} = U_{33}$;** $U_{12} = U_{23} = U_{13}$; Sb:*** $U_{12} = U_{13} = 0$;
R:****$U_{11} = U_{22} = U_{33}$

Table S2. Estimated carrier density calculated for each composition, using the Single Parabolic Band approximation, from the measured Seebeck coefficient. These values are not obtained from Hall effect measurements, but from the experimental Seebeck coefficient.

We have considered two different effective masses in the calculation, $1.5m_e$ and $1.7m_e$, common reported values in skutterudites.

Composition	Carrier density (10^{20} cm^{-3})
$La_{0.5}Co_4Sb_{11.6}Sn_{0.4}$	2.13 – 2.57
$Ce_{0.5}Co_4Sb_{11.6}Sn_{0.4}$	1.98 – 2.39
$Yb_{0.5}Co_4Sb_{11.6}Sn_{0.4}$	1.77 – 2.14
$La_{0.5}Co_4Sb_{12}$	6.07 – 7.33
$Ce_{0.5}Co_4Sb_{12}$	0.62 – 0.74
$Yb_{0.5}Co_4Sb_{12}$	1.77 – 2.14

Table S3. Refinement of the La occupancy factors from the SXRD data at different temperatures. To create this table, we have repeated the Rietveld refinements with released occupancy factor of the filler in the $La_{0.5}Co_4Sb_{11.6}Sn_{0.4}$ compound, although the occupancy factor and the displacement parameters are strongly related, making this analysis difficult.

The refinement of the other high-temperature structures was carried out by fixing the filling factors to those determined at RT, in order to avoid unwanted couplings with the displacement factors.

Temperature (K)	La occupancy (Phase 1)	La occupancy (Phase 2)
300	0.052(6)	0.33(2)
473	0.060(6)	0.36(2)
673	0.05(1)	0.35(2)
873	0.040(6)	0.34(2)
1073	0.054(7)	0.34(2)

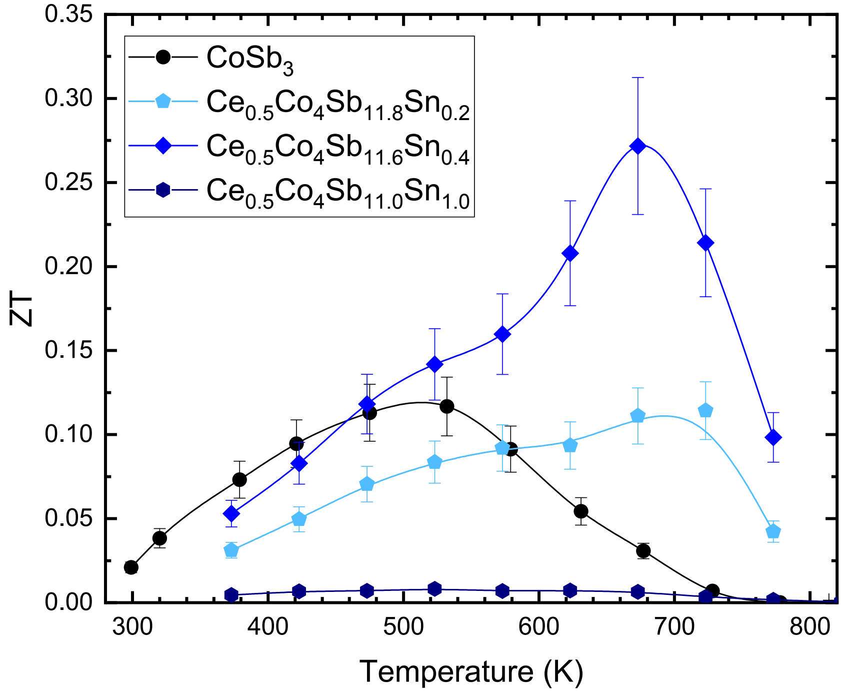

Figure S6. Temperature dependence of the figure of merit, ZT, of $CoSb_3$ and three Sn-doped compositions, $Ce_{0.5}Co_4Sb_{12-y}Sn_y$. The error bars are placed considering an error of ±15%.
